# Ferulic Acid Alleviates the Hepatotoxicity of Aflatoxin B1 on Broilers by Conjugating and Down-Regulating Chicken CYP1A5 and CYP2W1

**DOI:** 10.3390/vetsci13050476

**Published:** 2026-05-14

**Authors:** Xinghe Wang, Weiwei Li, Jianan Dai, Meng Jia, Lingfang Na, Wenyang Xu, Changde Wu, Mingchun Liu

**Affiliations:** 1 College of Animal Science and Veterinary Medicine, Shenyang Agricultural University, Shenyang 110866, China; wangxinghe@syau.edu.cn (X.W.); liweiwei93@syau.edu.cn (W.L.);; 2 Petsmate Shennong Hefeng Animal Hospital, Shenyang 110065, China

**Keywords:** ferulic acid, aflatoxin B1, CYP450 enzymes, broiler, liver damage

## Abstract

Aflatoxin B1 (AFB1) induces hepatocellular damage through its metabolite aflatoxin B1-8,9-epoxide (AFBO), which is produced in endoplasmic reticulum (ER) via cytochrome P450 (CYP450) enzymes. To investigate the effect of ferulic acid (FA) on AFB1-induced broiler liver damage, one-day-old Arbor Acres broilers were exposed to AFB1 (4 mg/kg) and treated with different doses of FA (60 mg/kg, 120 mg/kg, and 240 mg/kg) continuously for 28 days. The production performance, biochemical indicators, morphological changes, CYP450 enzymes’ expression in ER, interactions between small molecules and CYP450 enzymes, and CYP450 enzymes’ protein secondary structure were investigated. The results showed the following: (I) FA promoted broiler growth and reduced AFBO production. (II) AFB1-induced changes in serological indicators (AST, ALT, ALP, γ-GT, TBA, TG) and biochemical parameters (GST, SOD, MDA, ROS), which were reversed by FA. (III) AFB1-induced liver morphological changes and apoptosis were obviously alleviated by FA. (IV) AFB1-induced up-regulation of CYP1A5, CYP2A6, CYP2W1, and CYP3A4 in ER were reduced by FA. (V) The binding affinity of FA to CYP1A5 is lower than that of AFB1 to CYP1A5, and the binding affinity of FA to CYP2W1 is similar to that of AFB1 to CYP2W1. (VI) The contents of α helix, β sheet, β turn, and random coil in chicken CYP1A5 were 59.6%, 7.8%, 13.6%, and 19.0% respectively, and those in chicken CYP2W1 were 32.2%, 17.1%, 16.8%, and 33.9% respectively. In conclusion, FA can promote broiler growth and alleviate AFB1-induced hepatotoxicity via inhibiting and conjugating CYP450 enzymes, thus reducing AFBO formation and oxidative damage.

## Simple Summary

Aflatoxin B1 is a common pollutant in broiler feed, which can inhibit growth and immune system function, increase the susceptibility of chickens to infectious diseases, and increase mortality. Aflatoxin B1 needs to be bio-activated by cytochrome P450 enzymes to produce a highly toxic metabolite aflatoxin B1-8,9-epoxide, which can cause DNA damage, protein function disorder, and cell death. Ferulic acid is commonly found in plants and has a variety of biological activities. The role of ferulic acid in aflatoxin B1-induced liver damage in broiler remains unidentified. In this study, broilers were exposed to aflatoxin B1 and administrated with ferulic acid. The results proved that (i) adding ferulic acid to broilers’ feed can promote growth, (ii) ferulic acid can reduce the formation of aflatoxin B1-8,9-epoxide by inhibiting the production of cytochrome P450 enzymes in endoplasmic reticulum and conjugating with chicken cytochrome P450 enzymes, and (iii) ferulic acid can inhibit aflatoxin B1-induced oxidative damage by activating antioxidase, reducing reactive oxygen species, and inhibiting lipid peroxidation, all of which can effectively alleviate aflatoxin B1-induced liver injury. In conclusion, ferulic acid can improve broilers growth and prevent aflatoxin B1-induced broiler liver injury. It is of great significance to promote the healthy development of broiler breeding industry.

## 1. Introduction

Aflatoxin B1 (AFB1) is the most common pollutant in broiler feed. AFB1 contamination in feed inhibits the growth of broilers and damages the immune system, which can increase the susceptibility of chickens to infectious diseases and increase mortality. It is difficult to completely remove AFB1 from contaminated feed without reducing the nutrients. It is urgent to find an economic, effective, safe, and environmental-friendly method to reduce the toxicity of AFB1, which is of great significance to promote the healthy development of the poultry breeding industry.

AFB1 itself has no obvious toxicity. It must be bio-activated by cytochrome P450 (CYP450) isoenzymes to produce cytotoxicity and carcinogenesis. In human and animal bodies, AFB1 is activated by specific CYP450 enzymes to form AFM1, AFP1, AFQ1, AFL, and aflatoxin B1-8,9-epoxide (AFBO) [1,2]. AFBO is a highly toxic electrophilic compound that can covalently bind to biological macromolecules and cause cell oxidative damage. AFBO combines with DNA to form AFB1-N7-guanine (AFB1-DNA) adduct and then causes DNA breakage and DNA mutation. The binding of AFBO to intracellular proteins can cause abnormal protein folding and enzyme inactivation, which will lead to cell metabolic disorder. AFBO combines with albumin to form AFB1-albumin (AFB1-ALB) adduct. AFB1-ALB and AFB1-DNA can exist in blood and the liver for a long time. Because AFBO is an extremely unstable epoxide, its biological dose cannot be monitored in vivo. Therefore, AFB1-DNA and AFB1-ALB are used as biomarkers of human and animal exposure to AFB1. The highly toxic AFBO was produced by CYP450 enzymes in ER of hepatocytes [3]. Therefore, CYP450 enzymes could be the key targets to inhibit the hepatotoxicity of AFB1. High expressions of CYP1A5, CYP2A6, and CYP3A4 have been reported to play important roles in bio-activating AFB1 in chicken liver. However, the research on the effect of chicken CYP2W1 on AFB1 is still limited. This study will investigate the change of CYP2W1 in chicken liver exposed to AFB1.

FA is an important component of the plant cell wall. Therefore, it can be obtained from a variety of plants, especially some Chinese herbal medicines. FA has many biological activities and also has advantages of low cost, good safety, and a stable structure [4,5]. As an effective antioxidant, FA can play a hepatoprotective role in liver injury induced by alcohol, drugs, high-fat diet, γ-radiation, and cadmium [6]. Our team has reported that FA had a good effect on AFB1-induced liver injury in rats [7,8]. Up to now, the effect of FA in preventing and treating AFB1 poisoning in chickens is still unclear. This study will focus on chicken CYP450 enzymes to explore the potential of FA in protecting broilers against AFB1-induced liver damage and its mechanism.

## 2. Materials and Methods

### 2.1. Animals and Diets

One-day-old male Arbor Acres (AA) broilers were fed a basal diet (control group, C group), a diet with 4 mg/kg AFB1 (AFB1 exposure group, AFB1 group), a diet with 4 mg/kg AFB1 and 60 mg/kg FA (low dose FA administration group, L group), a diet with 4 mg/kg AFB1 and 120 mg/kg FA (medium dose FA administration group, M group), a diet with 4 mg/kg AFB1 and 240 mg/kg FA (high dose FA administration group, H group), and a diet with 240 mg/kg FA (FA control group, FA group) continuously for 28 days. Each group consisted of 15 broiler chicks, and each chick was individually housed. The ingredients and nutritional levels of the broiler basal diet are listed in Tables S1 and S2. AFB1 (purity ≥ 99.0%) was purchased from Pribolab Pte., Ltd., Singapore. FA (purity ≥ 99.0%) was purchased from Macklin Biochemical Technology Co., Ltd., Shanghai, China. AFB1 and FA were respectively dissolved by dimethyl sulfoxide (DMSO) and then equably mixed in the diet. Healthy one-day-old AA broiler chicks and the basal diet were purchased from Shenyang Huamei Livestock and Poultry Co., Ltd., Shenyang, China. It is an authorized commercial hatchery (Registration number: 210137000009141). After receiving the different diets for 28 days, broiler chickens were weighed and then euthanized with sodium pentobarbital and sacrificed by jugular vein bleeding. The blood obtained from the jugular vein was used for serological tests and detecting the content of AFB1-ALB adduct. The livers were immediately separated for biochemical tests, morphological tests, endoplasmic reticulum protein extraction, and detecting the content of AFB1-DNA adduct. The experimental protocols of this work were approved by the Shenyang Agricultural University Animal Care and Use Committee (Animal Ethics Procedures and Guidelines of the People’s Republic of China) prior to the initiation of this study. The approval number of welfare ethics of experimental animals is SNLL25022601.

### 2.2. Histopathological Examination and Cell Apoptosis Detection

As described in our former research [9], liver tissues of chickens were embedded in paraffin. Liver tissue sections (4 μm) were stained with hematoxylin and eosin (H&E). The histopathological changes were examined by 3 pathologists unaware of the experimental conditions. The liver histopathological score consists of 3 parts (score 0–12): first, a necrosis score (0–4) of 0 (no necrosis), 1 (necrosis < 10%), 2 (10–25% of necrosis), 3 (26–50% of necrosis), or 4 (necrosis > 50%); second, an inflammation score (0–4) of 0 (no inflammatory cell infiltration), 1 (inflammatory cell infiltration area < 10%), 2 (inflammatory cell infiltration area is 10–25%), 3 (inflammatory cell infiltration area is 26–50%), or 4 (inflammatory cell infiltration area > 50%); third, a hepatocellular ballooning score (0–4) of 0 (no ballooning), 1 (balloon cells < 10%), 2 (10–25% of balloon cells), 3 (26–50% of balloon cells), or 4 (balloon cells > 50%). The Terminal-deoxynucleoitidyl Transferase/(TdT-) Mediated Nick End Labeling (TUNEL) assay (Vazyme Biotech Co., Ltd., Nanjing, China) was used to detect apoptotic liver cells. The protocol has been reported in our previous research [7]. Five liver tissue sections, which were contributed by 5 birds in each group, were randomly chosen for calculating the proportion of apoptotic cells at 100× magnification. The optical microscope, fluorescence microscope, and pathological imaging system used in this study were all manufactured by Leica Microsystems (Wetzlar, Germany).

### 2.3. Ultrastructure Test

The protocol has been reported in our previous study [9]. Liver tissues were kept in 0.25% glutaraldehyde, fixed in 1% osmic acid, dehydrated in graded ethanol solutions and acetone, and embedded in epoxy resin. The ultra-thin sections (50–70 nm) of liver tissue were stained with 2% uranyl acetate and lead citrate. The ultrastructure of the liver cells was examined by a transmission electron microscope (Hitachi High-tech Co., Ltd., Tokyo H-7650, Japan). Three liver tissue ultra-thin sections, which were contributed by 3 birds in each group, were randomly chosen for the ultrastructural pathological score. The liver ultrastructural pathological score consists of 3 parts (score 0–12): first, an endoplasmic reticulum injury score (0–4) of 0 (no endoplasmic reticulum injury), 1 (endoplasmic reticulum injury < 10%), 2 (10–25% of endoplasmic reticulum injury), 3 (26–50% of endoplasmic reticulum injury), or 4 (endoplasmic reticulum injury > 50%); second, a mitochondrial damage score (0–4) of 0 (no mitochondrial damage), 1 (mitochondrial damage < 10%), 2 (10–25% of mitochondrial damage), 3 (26–50% of mitochondrial damage), or 4 (mitochondrial damage > 50%); third, a nuclear damage score (0–4) of 0 (no nuclear damage), 1 (nuclear damage < 10%), 2 (10–25% of nuclear damage), 3 (26–50% of nuclear damage), or 4 nuclear damage > 50%).

### 2.4. Serological Tests and Liver Biochemical Tests

Aspartate aminotransferase (AST), alanine aminotransferase (ALT), alkaline phosphatase (ALP), γ-glutamyltransferase (γ-GT), total bile acid (TBA), and triglyceride (TG) levels in broiler serum were measured with enzyme rate assay kits. Reactive oxygen species (ROS) were detected by the chemical fluorescence method with a reactive oxygen species assay kit. The malondialdehyde (MDA) level in rat liver was examined with a malondialdehyde assay kit. The activity of superoxide dismutase (SOD) and glutathione S transferase (GST) were respectively detected by the WST-1 method and colorimetric method with test kits. All the test kits were manufactured by Nanjing Jiancheng Bioengineering Company (Nanjing, China).

### 2.5. The Content of AFB1-ALB in Serum and the Content of AFB1-DNA in Liver

The content of AFB1-ALB adducts in chicken serum and the content of AFB1-DNA adduct in chicken liver were respectively detected by ELISA kits manufactured by Shanghai Jingkang Bioengineering Co., Ltd., Shanghai, China.

### 2.6. Western Blot

An endoplasmic reticulum protein extraction kit (BestBio Biotechnology Co., Ltd., Shanghai, China) was used to extract chicken hepatocellular endoplasmic reticulum protein. Western Blot experiment protocols have been reported in our former research [9]. The primary antibodies, CYP1A5 and β-actin, were manufactured by Proteintech Group, Inc., Rosemont, IL, USA. The primary antibodies, CYP2A6, CYP2W1, and CYP3A4, were manufactured by ImmunoWay Biotechnology Company, Plano, TX, USA. The horseradish peroxidase-conjugated antibodies (goat anti-rabbit and goat anti-mouse) were purchased from Elabscience Biotechnology Co., Ltd., Wuhan, China. Band intensities were measured with ImageJ software (version 1.54f, National Institutes of Health, Bethesda, MD, USA). Protein expressions of CYP1A5, CYP2A6, CYP2W1, and CYP3A4 in hepatocellular ER were normalized in relation to β-actin.

### 2.7. Endoplasmic Reticulum Proteomics Sequencing

The protocol of endoplasmic reticulum proteomics sequencing was provided by Personalbio Biotechnology Co., Ltd., Shanghai, China. Chicken hepatocellular ER protein samples were digested with Filter Aided Proteome Preparation. The peptides were desalted using a C18 Cartridge and lyophilized to remove moisture. The dried peptides were redissolved in 40 μL of dissolution buffer and accurately quantified by measuring the OD280 value. The samples were separated using an Easy nLC system equipped with a nanoflow high-performance liquid chromatography. The column was equilibrated with 95% mobile phase A (consisted of 0.1% formic acid in water). Samples were loaded via an autosampler onto a trapping column and then separated on an analytical column at a flow rate of 250 nL/min. The LC gradient was set as follows: at 0–50 min, mobile phase B [contained 0.1% formic acid in acetonitrile/water (84% acetonitrile)] increased linearly from 0 to 35%; at 50–58 min, mobile phase B rose to 100%; and at 58–60 min, mobile phase B was maintained at 100%. Following chromatographic separation, the samples were introduced into a Thermo Fisher mass spectrometer for a 140 min in-depth mass spectrometry analysis. Detection was performed in positive ion mode with the precursor ion scan range set at 300–1800 *m*/*z*. The full-scan MS resolution was 70,000 at *m*/*z* 200. After each full scan, 10 debris maps were collected according to specific parameters, with HCD as the MS^2^ activation type. The protein identification and quantification results were obtained by searching the chicken database using MaxQuant software (version 1.5.5.1). Based on the STRING database (http://string-db.org/ accessed on 20 January 2025), potential direct and indirect interactions among the target proteins were explored to generate a protein–protein interaction (PPI) network, which was subsequently analyzed. Additionally, GO annotation analysis of the target protein set was conducted using ClueGO software (version 2.5.9).

### 2.8. Molecular Docking of FA on Chicken CYP450 Enzymes’ Binding Sites

The coding sequences of the target chicken CYP450 enzymes were obtained based on the following reference sequences from the NCBI database. Chicken CYP1A5: The coding region corresponds to amino acids (aa) 154-1740 (GenBank accession: NM_205146.3). Chicken CYP2A6: The coding region spans aa 40-1515 (GenBank accession: NM_001001616.2). Chicken CYP2W1: The coding region covers aa 57-1373 (GenBank accession: XM_015294328.4). Chicken CYP3A4: The coding region includes aa 42-1573 (GenBank accession: NM_001329508.2). ProtParam was employed to determine fundamental parameters including the relative molecular weight, hydrophobicity/hydrophilicity profile, and isoelectric point for chicken CYP1A5, CYP2W1, CYP2A6, and CYP3A4 enzymes. TMHMM Server v.2.0 was utilized for transmembrane domain prediction. SignalP 4.1 Server was applied for signal peptide identification. SWISS-MODEL online platform was used for tertiary structure prediction. Protein model quality was comprehensively evaluated using Procheck software. The molecular structure of FA was retrieved from the PubChem database. AutoDock software (version 4.2.6) facilitated systematic docking simulations between FA and each CYP450 isoform (chicken CYP1A5, CYP2W1, CYP2A6, and CYP3A4). Discovery Studio 4.5 Client software was used to visualize the docking results. The molecular docking parameters and model quality metrics are provided in Figure S3.

### 2.9. Binding Affinity of FA and AFB1 to Chicken CYP450 Enzymes

The binding affinity of FA and AFB1 to chicken CYP1A5 and chicken CYP2W1 were detected with surface plasmon resonance (SPR) technology. The operating procedure and the program setting have been reported in our previous study [8]. Chicken CYP1A5 protein and chicken CYP2W1 protein expressed by Escherichia coli (Supplementary Figure S1) were coupled to Series S Sensor chip CM7 (Cytiva, GE Healthcare BioSciences AB, Uppsala, Sweden). The binding affinity of small molecules (FA and AFB1) to chicken CYP450 enzymes (CYP1A5 and CYP2W1) was detected by the multi-cycle dynamic detection method. The results were shown as the equilibrium dissociation constant between small molecules (FA and AFB1) and chicken CYP450 enzymes (CYP1A5 and CYP2W1). The surface plasmon resonance instrument Biacore S200 and the analyzing software Biacore Insight were manufactured by Cytiva, Uppsala, Sweden.

### 2.10. The Secondary Structures of Chicken CYP1A5 and CYP2W1 Proteins Were Detected by Circular Dichroism Spectrometer

Prokaryotically expressed and purified chicken CYP1A5 and CYP2W1 proteins were freeze-dried and then redissolved with PBS (pH = 7.4). The protein concentrations of chicken CYP1A5 and chicken CYP2W1 were respectively adjusted to 0.5 mg/mL and then used to detect the secondary structure. The secondary structures of chicken CYP1A5 and CYP2W1 proteins were determined by MOS-500 multifunctional circular dichroism spectrometer (Biologic Science Instruments, Grenoble, France).

### 2.11. Statistical Analysis

The experiments of the serological tests (ALT, AST, ALP, γ-GT, TBA, TG), liver biochemical tests (GST, SOD, MDA, ROS), AFB1-DNA detection, AFB1-ALB detection, endoplasmic reticulum proteomics sequencing, and protein relative expressions of CYP1A5, CYP2A6, CYP2W1, and CYP3A4 were repeated at least 3 times. The apoptotic cell count was repeated 5 times. The results of the body weight gain, serological tests, liver biochemical tests, AFB1-DNA concentration, AFB1-ALB concentration, endoplasmic reticulum proteomics sequencing, apoptotic cell count, and the relative expressions of CYP1A5, CYP2A6, CYP2W1, and CYP3A4 proteins were expressed as the mean ± standard deviation. SPSS version 21.0 was used for data analyses. Two-way ANOVA followed by Tukey’s post hoc test was used to compare the quantitative data differences among the groups, which included the data of body weight gain, serological tests, liver biochemical tests, AFB1-ALB content in serum, AFB1-DNA content in liver, endoplasmic reticulum proteomics sequencing, and CYP1A5, CYP2A6, CYP2W1, and CYP3A4 protein relative expressions. The Shapiro–Wilk test was performed to verify the normal distribution of data. Levene’s test was applied to check the homogeneity of variances across groups.

## 3. Results

### 3.1. FA Promoted the Growth of Broilers

**Figure 1:**
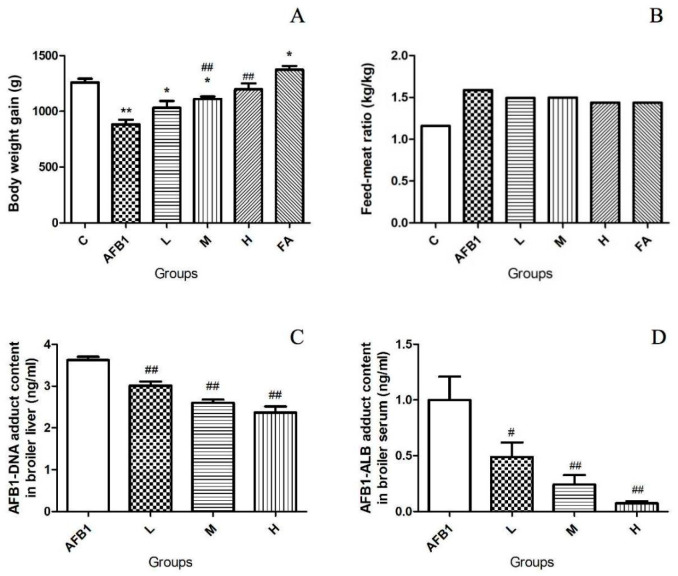
The influence of AFB1 exposure and FA administration on body weight gain, feed–meat ratio, and AFB1 adducts contents in broilers. The body weight gain changes of broilers in different groups are shown in panel (**A**). The feed–meat ratio changes of broilers in different groups are shown in panel (**B**). The effect of FA on the content of AFB1-DNA adduct in chicken liver is shown in panel (**C**). The effect of FA on the content of AFB1-ALB adduct in chicken serum is shown in panel (**D**). Asterisks * represent a statistical difference compared to the control group; # represents a statistical difference compared to the AFB1 group. * 0.01 < *p* < 0.05, ** *p* < 0.01; # 0.01 < *p* < 0.05, ## *p* < 0.01.

As shown in Figure 1A, after 28 days of feeding with different diets, the body weight gain of broilers in the AFB1 group was significantly lower than that of chickens in the control group (*p* < 0.01). The body weight gain of broilers in the L group was higher than that in the AFB1 group, but there was no statistical significance. The body weight gain of broilers in the M group and H group was significantly higher than in the AFB1 group (*p* < 0.01). The body weight gain of broilers in the FA group was higher than in the control group (*p* < 0.05). As shown in Figure 1B, the feed–meat ratio of broilers in the L group, M group, H group, and FA group was all lower than in the AFB1 group but higher than in the control group.

### 3.2. FA Inhibited the Formation of AFB1 Adducts

The results in Figure 1C and Figure 1D show that the content of AFB1-DNA adduct in chicken liver was reduced by 16.9%, 28.2%, and 34.5%, respectively, in the L group, M group, and H group compared to the AFB1 group (*p* < 0.01). The content of AFB1-ALB adduct in serum was reduced by 51.1%, 75.8%, and 92.5%, respectively, in the L group, M group, and H group in comparison with the AFB1 group (*p* < 0.01).

### 3.3. FA Alleviated Broiler Liver Damage Induced by AFB1

**Figure 2:**
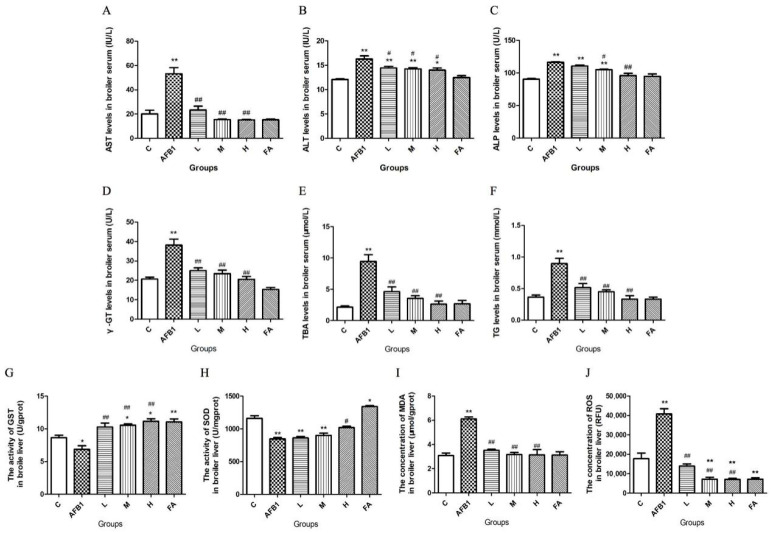
The influence of AFB1 exposure and FA administration on serological indicators and liver biochemical parameters. The levels of AST, ALT, ALP, γ-GT, TBA, and TG in chicken serum are respectively shown in panels (**A**–**F**). The activities of GST and SOD in chicken liver are respectively shown in panels (**G**,**H**). The contents of MDA and ROS in broiler liver are separately shown in panels (**I**,**J**). Asterisks * represent a statistical difference compared to the control group; # represents a statistical difference compared to the AFB1 group. * 0.01 < *p* < 0.05, ** *p* < 0.01; # 0.01 < *p* < 0.05, ## *p* < 0.01.

As shown in Figure 2, AFB1 exposure increased the levels of AST, ALT, ALP, γ-GT, TBA, and TG in chicken serum (*p* < 0.01), inhibited the activities of GST (*p* < 0.05) and SOD (*p* < 0.01) in chicken liver, and up-regulated the contents of MDA and ROS in chicken liver (*p* < 0.01). FA treatment dose-dependently reduced the AST, ALT, ALP, γ-GT, TBA, TG levels in serum (*p* < 0.05 or *p* < 0.01), promoted the activities of antioxidase GST (*p* < 0.01) and SOD (*p* < 0.05 in the H group), and reduced the concentrations of the oxidative damage biomarkers MDA and ROS (*p* < 0.01). Compared with the control group, the GST activity and SOD activity in the FA group were both increased (*p* < 0.05 or *p* < 0.01), and the content of ROS in the FA group was significantly decreased (*p* < 0.01).

**Figure 3:**
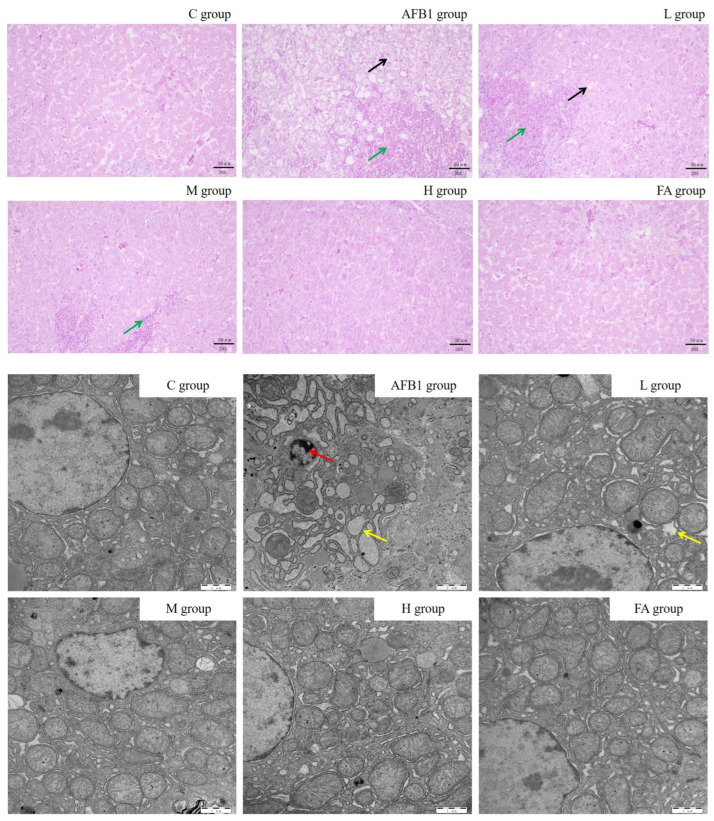
The histopathological changes in chicken liver and the ultrastructure changes in chicken hepatocytes. Liver sections from C, AFB1, L, M, H, and FA groups were stained by hematoxylin and eosin (H&E) and examined under light microscopy. The images are displayed at 200× the original magnification. Cell degeneration and necrosis are pointed out by a black arrow. Inflammatory cell infiltration is pointed out by a green arrow. The images reveal chicken hepatocellular ultrastructure at 20,000× the original magnification. Chromatin edge aggregation in AFB1 group is pointed out by a red arrow. Endoplasmic reticulum dilated and swollen in AFB1 group and L group is pointed out by a yellow arrow.

**Figure 4:**
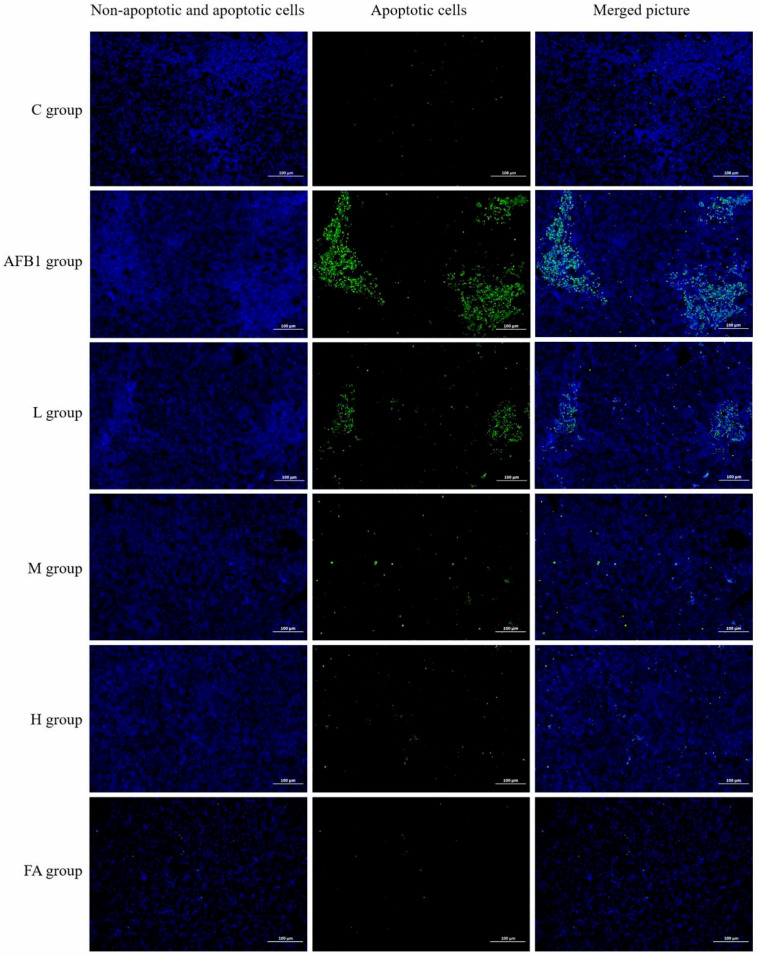
Cell apoptosis in broiler liver. Chicken liver sections were tested by TUNEL FITC apoptosis detection kit. The apoptotic cells showed green fluorescence. The non-apoptotic cells showed blue fluorescence. The pictures are displayed at 100× magnification.

The results in Figure 3 and Table 1 and Table 2 show that the liver tissue structure of broilers in the C group, H group, and FA group was normal, with clear central veins, hepatic cords, hepatic sinusoidal, and uniform staining. In the AFB1 group, the necrosis score, inflammation score, ballooning score, and total histopathological score were all higher than in the control group (*p* < 0.01). In the L group, these scores were higher than in the control group (*p* < 0.01) and lower than in the AFB1 group (*p* < 0.01). In the M group, the necrosis score was much lower than in the AFB1 group (*p* < 0.01). The inflammation score, ballooning score, and total histopathological score were higher than in the control group (*p* < 0.01) and lower than in the AFB1 group (*p* < 0.01). The ultra-structure detection of liver cells found that the hepatocytes in the C, H, and FA groups had a complete structure, regular nucleus shape, uniform distribution of chromatin, obvious nucleolus, and a clear structure of major organelles such as mitochondria and ER. In the AFB1 group, the endoplasmic reticulum injury score, mitochondrial damage score, nuclear damage score, and liver ultrastructural pathological score were higher than in the C group (*p* < 0.01). In the L group, these scores were higher than in the C group (*p* < 0.01) and lower than in the AFB1 group (*p* < 0.01). In the M group, the endoplasmic reticulum injury score was lower than in the AFB1 group (*p* < 0.01). The mitochondrial damage score, nuclear damage score, and liver ultrastructural pathological score were higher than in the control group (*p* < 0.05 or *p* < 0.01) and lower than in the AFB1 group (*p* < 0.01). The results of apoptotic cell detection (Figure 4 and Table 3) showed that the percentage of apoptotic liver cells in the AFB1 group was 36.9 times higher than in the control group (*p* < 0.01). The percentage of apoptotic liver cells in the L group was significantly lower than in the AFB1 group (*p* < 0.01) and 18.5 times higher than in the control group (*p* < 0.01). The proportion of apoptotic cells in the M group was 5.7 times lower than in the AFB1 group (*p* < 0.01) and 5.6 times higher than in the control group (*p* < 0.05). The proportion of apoptotic cells showed no significant difference in the C group, H group, and FA group.

### 3.4. AFB1-Induced High Expression of CYP450 Enzymes in Endoplasmic Reticulum Was Inhibited by FA

**Figure 5:**
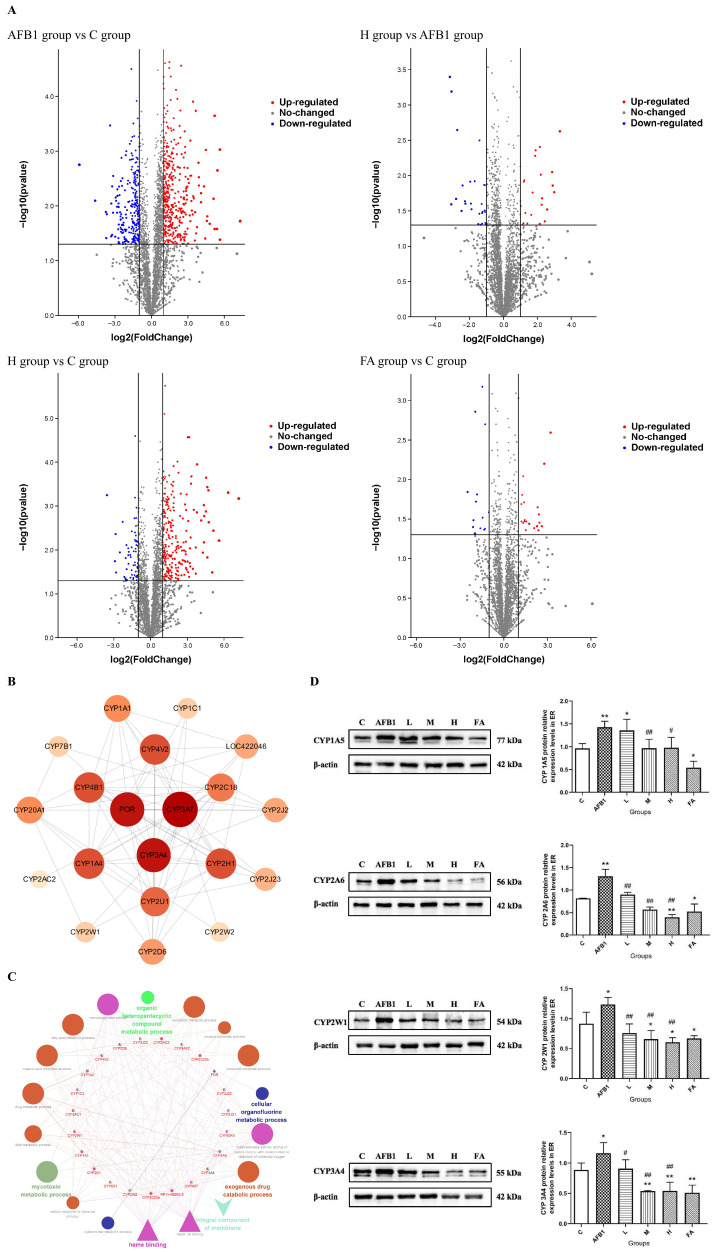
The influence of AFB1 exposure and FA administration on CYP450 enzymes expression in endoplasmic reticulum of broiler hepatocytes. Panel (**A**): Volcano plots of endoplasmic reticulum proteome sequencing results in hepatocytes of broilers from group C, group AFB1, group H, and group FA. Red dots represent up-regulated proteins, and blue dots represent down-regulated proteins. Panel (**B**): PPI network diagram of differentially expressed CYP450 enzymes in endoplasmic reticulum proteome sequencing of broiler hepatocytes in groups C, AFB1, H, and FA. Panel (**C**): GO signaling pathway analysis of the differentially expressed CYP450 enzymes in endoplasmic reticulum proteome sequencing of broiler hepatocytes in C, AFB1, H, and FA groups. Panel (**D**): The protein relative expression of chicken CYP1A5, CYP2A6, CYP2W1, and CYP3A4 in hepatocellular endoplasmic reticulum. Asterisks * represent a statistical difference compared to the control group; # represents a statistical difference compared to the AFB1 group. * 0.01 < *p* < 0.05, ** *p* < 0.01; # 0.01 < *p* < 0.05, ## *p* < 0.01.

The screening criteria for differentially expressed proteins were set as follows: proteins with a fold change (FC) ≥ 2.0 were defined as up-regulated, while those with FC ≤ 0.5 were defined as down-regulated. A statistical significance of *p* < 0.05 and coefficient of variation (CV) < 30% within biological replicates were required for screening differentially expressed proteins. As shown in Figure 5, the sequencing results of the endoplasmic reticulum proteome showed that there are 669 proteins changed in the AFB1 group compared to the control group, 49 proteins changed in the H group compared to the AFB1 group, and 39 proteins changed in the FA group compared to the C group. The sequencing results of the endoplasmic reticulum proteome found that, although 20 kinds of CYP450 enzymes had obvious changes, only CYP2W1 and CYP3A4 were up-regulated significantly in the AFB1 group compared with the C group and were down-regulated in the H group compared with the AFB1 group, which means chicken CYP2W1 and CYP3A4 may be important targets for FA to reduce the hepatotoxicity of AFB1. Combined with the sequencing results of the endoplasmic reticulum proteome and previous literature reports, CYP1A5, CYP2W1, CYP2A6, and CYP3A4 were selected for verification with Western Blot. The results showed that the protein relative expression of CYP1A5 and CYP2A6 in hepatocellular ER was extremely significantly increased in the AFB1 group compared to the control group (*p* < 0.01). The protein expression of CYP2W1 and CYP3A4 in ER was increased in the AFB1 group compared to the control group (*p* < 0.05). In the L group, M group, and H group, the protein expression levels of CYP1A5, CYP2A6, CYP2W1, and CYP3A4 in ER were dose-dependently decreased compared to the AFB1 group (*p* < 0.05 or *p* < 0.01). The CYP1A5 protein expression level in the hepatocellular ER of the FA group was statistically lower than in the control group (*p* < 0.05). The CYP2A6 protein expression in the hepatocellular ER of the H group and FA group was lower than in the control group (*p* < 0.05 or *p* < 0.01). CYP2W1 and CYP3A4 protein expression in the hepatocellular ER of the M group, H group and FA group were lower than in the C group (*p* < 0.05 or *p* < 0.01).

### 3.5. Molecular Docking Predicted That FA Had Strong Affinity with Chicken CYP1A5, CYP2A6, CYP2W1, and CYP3A4

**Figure 6:**
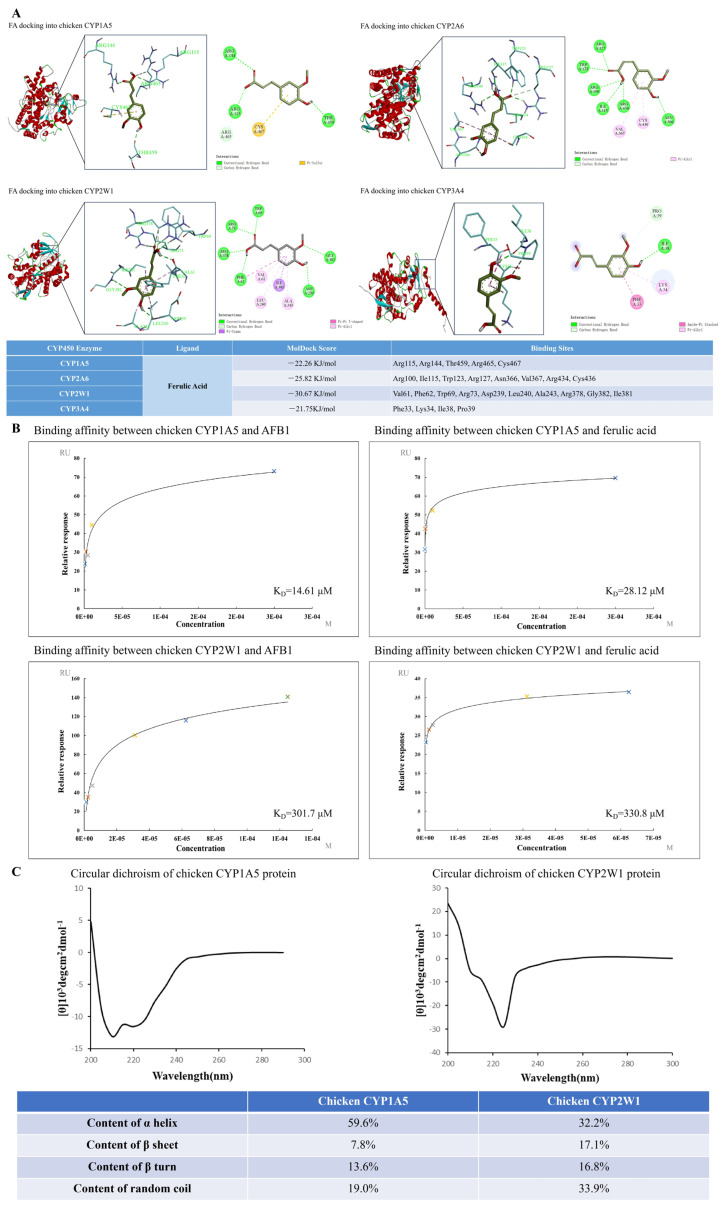
The binding affinity between small molecules (FA and AFB1) and chicken CYP450 enzymes and the secondary structure of chicken CYP450 enzymes. Panel (**A**): The predicted binding models of FA docking into chicken CYP1A5, chicken CYP2A6, chicken CYP2W1, and chicken CYP3A4. Panel (**B**): The binding affinity between small molecules (FA and AFB1) and macromolecules (chicken CYP1A5 and chicken CYP2W1) detected by SPR. Panel (**C**): The secondary structure characteristics of chicken CYP1A5 and chicken CYP2W1 proteins detected with circular dichroism spectrometer.

The results of molecular docking (Figure 6A and Figure S2) showed that five amino acid sites in chicken CYP1A5 could be docked by FA. The binding energy of FA to chicken CYP1A5 was predicted as −22.26 KJ/mol. There are eight amino acid sites in chicken CYP2A6 that can be docked by FA, and the binding energy of FA to chicken CYP2A6 is predicted as −25.82 KJ/mol. Chicken CYP2W1 has 10 amino acid sites that can be conjugated by FA, and the binding energy between FA and chicken CYP2W1 is predicted as −30.67 KJ/mol. Four binding sites that can be docked by FA were predicted in chicken CYP3A4. The binding energy between FA and chicken CYP3A4 was predicted as −21.75 KJ/mol. Molecular docking simulation showed that the binding energy of FA to chicken CYP450 enzymes was as follows: CYP2W1 > CYP2A6 > CYP1A5 > CYP3A4. Arachidonic acid (ARA) is the endogenous substrate of chicken CYP1A5, CYP2A6, CYP2W1, and CYP3A4. The binding energy of ARA to chicken CYP1A5, CYP2A6, CYP2W1, and CYP3A4 was −22.84 KJ/mol, −30.07 KJ/mol, −25.45 KJ/mol, and −28.07 KJ/mol, respectively.

### 3.6. The Actual Binding Affinity of FA and AFB1 to Chicken CYP1A5 and Chicken CYP2W1

The binding affinity of FA and AFB1 to human CYP2A6 has been investigated in our previous study [7,8]. According to the molecular docking results, chicken CYP1A5 and chicken CYP2W1 showed strong binding energy with FA. So, chicken CYP1A5 and chicken CYP2W1 were selected to further investigate their actual binding affinity with FA and AFB1. The technology of SPR was used to detect the actual binding affinity of FA and AFB1 to chicken CYP1A5 and chicken CYP2W1. As shown in Figure 6B, the equilibrium dissociation constant between AFB1 and chicken CYP1A5 was 14.61 µM. The equilibrium dissociation constant between FA and chicken CYP1A5 was 28.12 µM. The equilibrium dissociation constant between AFB1 and chicken CYP2W1 was 301.7 µM. The equilibrium dissociation constant between FA and chicken CYP2W1 was 330.8 µM.

### 3.7. The Secondary Structure of Chicken CYP1A5 and Chicken CYP2W1 Showed Different Characteristics

The secondary structure of chicken CYP1A5 and chicken CYP2W1 was detected with circular dichroism chromatograph. The results in Figure 6C show that the content of α helix was 59.6% in chicken CYP1A5 and was 32.2% in chicken CYP2W1. The proportion of β sheet was 7.8% in chicken CYP1A5 and was 17.1% in chicken CYP2W1. The percentage of β turn was 13.6% in chicken CYP1A5 and was 16.8% in chicken CYP2W1. The content of random coil was 19.0% in chicken CYP1A5 and was 33.9% in chicken CYP2W1.

## 4. Discussion

AFB1 is the most common pollutant in chicken feed, which has caused huge economic loss to broiler breeding industry. In recent years, many studies have proved that FA has a protective effect on hepatocytes in liver injury induced by many different factors. This study explored the potential of FA in protecting broilers against AFB1-induced liver injury.

Our data in Figure 1 prove that AFB1 can significantly inhibit the growth of broilers. FA administration alleviated the poor growth of broilers caused by AFB1 in a dose-dependent manner. Broilers in the FA group had statistically more body weight gain than broilers in the control group, which proved that adding FA to broiler feed significantly promoted the growth of broilers. Although both AFB1 exposure and FA administration increased the feed–meat ratio compared to the control group, FA significantly promoted the growth of broilers. This finding is of great significance for the application of FA in poultry healthy breeding. AFB1 must be bio-activated by CYP450 isoenzymes to produce AFBO, which has cytotoxicity and carcinogenesis. The content of AFB1 adducts (AFB1-DNA and AFB1-ALB) is used to evaluate the level of AFBO. Detection of AFB1 adducts proved that adding FA to broiler feed inhibited the production of AFBO.

When hepatocytes are damaged, AST and ALT leak into the blood from cytoplasm and mitochondria. ALP is synthesized in bone, then transported to the liver through blood, and finally excreted by biliary system. γ-GT and TBA in serum primarily come from the hepatobiliary system. When liver cells are damaged, their ability to synthesize, disintegrate, and export TG will be reduced, resulting in TG accumulation in the blood. In Figure 2, the increase in serum ALT, AST, ALP, γ-GT, TBA, and TG in AFB1 group give evidence of serious damage to the hepatobiliary system, while the decrease in ALT, AST, ALP, γ-GT, TBA, and TG in the FA administration groups compared to the AFB1 group proved that FA shows good performance in alleviating AFB1-induced broiler hepatobiliary injury, and the effect of FA on the hepatotoxicity of AFB1 showed a dose-dependent manner. GST and SOD are both antioxidases. GST is the rate-limiting enzyme of AFB1 detoxification metabolism [1]. It can catalyze the combination of glutathione (GSH) and AFBO to form a nontoxic adduct AFB1-GSH, which is excreted through urine. The function of SOD is to scavenge oxygen free radicals. ROS are oxygen free radicals, which can attack DNA, oxidize proteins, and induce lipid peroxidation. MDA is the main product of lipid peroxidation. The content of MDA is positively correlated with the intensity of lipid peroxidation. The changes in antioxidase activities and oxidative damage biomarkers indicated that FA is an active antioxidant, and it can protect broiler liver from the toxicity of AFB1 by promoting the activation of GST and SOD, reducing the formation of ROS, and inhibiting lipid peroxidation.

The results of AFBO formation detection, serological tests, detection of indexes correlated to oxidative stress, and morphological examinations (Figure 1, Figure 2 and Figure 3 and Table 1, Table 2 and Table 3) jointly proved that reducing AFBO formation and resisting oxidative damage were important ways for FA to prevent AFB1-induced liver cell death in broilers. Further, the protective effect of FA on broiler hepatocytes was dose-dependent.

In different kinds of poultry, the CYP450 enzymes involved in activating AFB1 are different. In duck, CYP1A1, CYP1A2, CYP2A6 and CYP3A4 orthologs are involved in the bio-activation of AFB1 [10]. In turkey, chicken, and quail, CYP1A1 and CYP2A6 orthologs are involved in AFBO production [11,12]. In vitro studies found that the CYP1A5 ortholog could activate AFB1 to produce AFBO and AFM1 [5,13]. In vivo, studies reported that CYP1A1, CYP1A2, CYP2A6, and CYP3A4 orthologs are involved in the bio-activation of AFB1 in chicken [14,15]. The results of ER proteomics sequencing and Western Blot verification (Figure 5) in our study proved that FA administration could down-regulate AFB1-induced high production of CYP1A5, CYP2A6, CYP2W1, and CYP3A4 in broiler liver. This phenomenon provided a hypothesis that chicken CYP1A5, CYP2A6, CYP2W1, and CYP3A4 were the targets of FA.

Chicken CYP1A5 is a homologous enzyme to mammalian CYP1A2. It has been reported that, when the concentration of AFB1 in turkey liver was 0.1 μM, CYP1A5 accounted for 98% of the total amount of AFBO formation [12]. In vitro research also found that chicken CYP1A5 played an important role in bio-activating AFB1 [5]. Our study provided in vivo and in vitro evidence that CYP1A5 could be conjugated and induced by AFB1 in broiler liver, and FA could reduce the hepatotoxic effect of AFB1 through inhibiting the production of CYP1A5 in broiler hepatocellular ER and directly conjugating with chicken CYP1A5. Many studies have reported that AFB1 causes broiler liver injury through up-regulating CYP2A6 [16,17,18]. Our study also proved this. In this research, FA was proved to have the function of reducing the hepatotoxicity of AFB1 by inhibiting the production of CYP2A6 in ER of broiler hepatocytes. CYP2W1 was first cloned from a human hepatocellular carcinoma cell line [19]. CYP2W1 is considered a marker of specific cancer. It has been found that CYP2W1 has catalytic activity in the process of transforming AFB1 into cytotoxic products in human embryonic kidney cells (HEK293) [20]. Different from classic endoplasmic reticulum membrane CYP450 enzymes (such as CYP1A5, CYP2A6, etc.), CYP2W1 is atypically located in ER. The CYP2W1 protein topologically reverse binds to glycosyltransferase in the lumen space of ER. This special localization leads part of the CYP2W1 protein from ER to the cell membrane. It has been proved that approximately 10% of CYP2W1 is located on cell membrane. The topological reverse orientation of CYP2W1 makes it widely distributed in the hepatocyte membrane system [21]. Whether CYP2W1 is expressed in broiler liver, the protein structure of chicken CYP2W1, and whether it can interact with AFB1 in broiler are still unknown. In this study, the expression changes of CYP2W1 protein in broiler hepatocellular ER in different groups and the binding affinity of AFB1 to chicken CYP2W1 not only confirmed the existence of CYP2W1 in broiler hepatocytes but also provided evidence that CYP2W1 had direct interaction with AFB1. In addition, the reduced expression of CYP2W1 in the M, H, and FA groups and the binding affinity of FA to chicken CYP2W1 together demonstrated that FA could prevent AFB1-induced CYP2W1 high production and directly bind to CYP2W1.

The equilibrium dissociation constants between small molecules (FA and AFB1) and chicken CYP450 enzymes (CYP1A5 and CYP2W1) give evidence that the order of binding capacity from large to small is AFB1 with CYP1A5 > FA with CYP1A5 > AFB1 with CYP2W1 ≈ FA with CYP2W1. These results indicated that chicken CYP1A5 and CYP2W1 both played an important role in interacting with AFB1. We can speculate that FA can regulate chicken CYP1A5 and CYP2W1 via direct combination. However, the binding strengths are different. The α helix is of high significance to the stability of protein structure and the implementation of protein function. The binding process between enzyme and substrate depends on the α helix [22]. The content of α helix in chicken CYP1A5 was much higher than that in chicken CYP2W1. We speculate that this is the reason why the binding affinity between CYP1A5 and small molecules (FA and AFB1) is stronger than that between CYP2W1 and small molecules (FA and AFB1). A high proportion of β sheet, β turn and random coil in chicken CYP2W1 can increase the exposure of its functional sites. In silico analysis in our study proved that chicken CYP2W1 had more binding sites than chicken CYP1A5, CYP2A6, and CYP3A4 when docked to FA. We speculate that this may be due to the high proportion of β sheet, β turn and random coil in chicken CYP2W1.

When human hepatocytes were exposed to 25–500 μM AFB1, CYP3A4 was involved in 79–95% of AFBO formation. This phenomenon indicated that CYP3A4 was an important enzyme responsible for the epoxidation of AFB1 in human hepatocytes [23]. In recent research, it has been reported that CYP3A4 played an important role in catalyzing the epoxidation of AFB1 in broilers [24]. So, inhibiting the expression of CYP3A4 could be an effective way to alleviate AFB1-induced liver damage. The results of our study proved that AFB1 could induce the high production of CYP3A4 in broiler liver, and FA could alleviate AFB1-induced broiler liver injury via reducing the production of CYP3A4 in hepatocellular ER.

Studies in human cell lines and rats reported that FA alone can inhibit the expression of several key CYP450 isoforms (including CYP3A4, CYP1A1/2, and CYP2E1), mainly through direct binding to the enzyme active sites [8] and reducing oxidative stress-dependent CYP450 up-regulation [25,26]. In our study, FA alone inhibited the protein expression of CYP1A5, CYP2A6, CYP2W1, and CYP3A4 compared to the control group (*p* < 0.05 or *p* < 0.01). Our results proved the inhibitory effect of FA on chicken CYP450 enzymes via direct binding to the active sites of chicken CYP450 enzymes and inhibiting oxidative stress. The results of molecular docking showed that compared with the endogenous substrate ARA, the binding energy of FA to chicken CYP1A5, CYP2A6, CYP2W1, and CYP3A4 was similar to their endogenous substrates. It means FA has strong affinity for the above-mentioned chicken CYP450 enzymes. Chicken CYP2W1 has the most active site residues that can be docked by FA. They are Val 61, Phe62, Trp69, Arg73, Asp239, Leu240, Ala243, Arg378, Ile381, and Gly382. The types of molecular interaction between FA and CYP2W1 are the most abundant. The molecular force types include a conventional hydrogen bond, a carbon hydrogen bond, Pi-Pi T-shaped, Pi-Sigma, and Pi-Alkyl. FA bound to chicken CYP2W1 with abundant active sites and diverse interaction modes, indicating that it has high binding stability, strong affinity and good target specificity. Multiple synergistic intermolecular forces jointly maintained the stable binding conformation.

## 5. Conclusions

This research proves that (i) adding FA to broiler feed can promote broiler growth, (ii) FA can reduce the formation of AFBO by inhibiting the production of CYP450 enzymes (CYP1A5, CYP2A6, CYP2W1, and CYP3A4) in endoplasmic reticulum and competing with AFB1 to conjugate with CYP450 enzymes (CYP1A5 and CYP2W1), (iii) FA can inhibit AFB1-induced oxidative damage through activating antioxidase (GST and SOD), reducing ROS, and inhibiting lipid peroxidation, all of which effectively alleviate AFB1-induced liver injury. This study concludes that FA incorporation in feed not only can improve broiler growth but also is a potential method for preventing AFB1-induced broiler liver injury.

## Figures and Tables

**Table 1:** Liver histopathological score.

Groups	Necrosis Score	Inflammation Score	Ballooning Score	Histopathological Score
C group	0 ± 0	0 ± 0	0 ± 0	0 ± 0
AFB1 group	3.67 ± 0.58 **	4 ± 0 **	3.33 ± 0.58 **	11 ± 1 **
L group	1.33 ± 0.58 **^;##^	3 ± 0 **^;##^	2.33 ± 0.58 **^;##^	6.67 ± 1.15 **^;##^
M group	0.33 ± 0.58 ^##^	1.33 ± 0.58 **^;##^	1 ± 0 **^;##^	2.67 ± 1.15 **^;##^
H group	0 ± 0 ^##^	0 ± 0 ^##^	0 ± 0 ^##^	0 ± 0 ^##^
FA group	0 ± 0	0 ± 0	0 ± 0	0 ± 0

^1^ The data represent means ± standard deviation. Asterisks * represent a statistical difference compared to the control group; # represents a statistical difference compared to the AFB1 group. * 0.01 < *p* < 0.05, ** *p* < 0.01; # 0.01 < *p* < 0.05, ## *p* < 0.01.

**Table 2:** Liver ultrastructural pathological score.

Groups	Endoplasmic Reticulum Injury Score	Mitochondrial Damage Score	Nuclear Damage Score	Liver Ultrastructural Pathological Score
C group	0 ± 0	0 ± 0	0 ± 0	0 ± 0
AFB1 group	4 ± 0 **	4 ± 0 **	3.67 ± 0.58 **	11.67 ± 0.58 **
L group	1 ± 0 **^;##^	2.33 ± 0.58 **^;##^	2.33 ± 0.58 **^;##^	5.67 ± 1.15 **^;##^
M group	0.33 ± 0.58 ^##^	1 ± 0 **^;##^	0.67 ± 0.58 *^;##^	2 ± 0 **^;##^
H group	0 ± 0 ^##^	0 ± 0 ^##^	0 ± 0 ^##^	0 ± 0 ^##^
FA group	0 ± 0	0 ± 0	0 ± 0	0 ± 0

^1^ The data represent means ± standard deviation. Asterisks * represent a statistical difference compared to the control group; # represents a statistical difference compared to the AFB1 group. * 0.01 < *p* < 0.05, ** *p* < 0.01; # 0.01 < *p* < 0.05, ## *p* < 0.01.

**Table 3:** Percentage of apoptotic liver cells.

Groups	Percentage of Apoptotic Liver Cells (%)
C group	1.28 ± 0.15
AFB1 group	48.45 ± 2.76 **
L group	24.97 ± 1.15 **^;##^
M group	8.45 ± 2.61 *^;##^
H group	1.27 ± 0.26 ^##^
FA group	0.99 ± 0.08

^1^ Fifteen pictures were randomly selected for counting the number of apoptotic cells in each group. The data represent means ± standard deviation. Asterisks * represent a statistical difference compared to the control group; # represents a statistical difference compared to the AFB1 group. * 0.01 < *p* < 0.05, ** *p* < 0.01; # 0.01 < *p* < 0.05, ## *p* < 0.01.

## Data Availability

The original contributions presented in this study are included in the article/Supplementary Material. Further inquiries can be directed to the corresponding authors.

## Supplementary Materials

The following supporting information can be downloaded at https://www.mdpi.com/article/10.3390/vetsci13050476/s1, Figure S1. The results of chicken CYP1A5 recombinant protein purification and identification and the results of chicken CYP2W1 recombinant protein purification and identification. Figure S2. The predicted binding models of endogenous substrate (arachidonic acid) docking into chicken CYP1A5, CYP2A6, CYP2W1, and CYP3A4. Figure S3. The model quality metrics and molecular docking parameters. Table S1. The ingredients of broiler basal diet. Table S2. The nutritional levels of broiler basal diet. Table S3. The raw data of broiler chickens’ body weight gain in different groups. Table S4. The raw data of AFB1-DNA concentration in broiler livers and AFB1-ALB concentration in broiler serum. Table S5. The raw data of serological tests and liver biochemical tests. Table S6. The raw data of CYP450 protein expression in endoplasmic reticulum.
