# Supplementary material for: Ferulic Acid Alleviates the Hepatotoxicity of Aflatoxin B1 on Broilers by Conjugating and Down-Regulating Chicken CYP1A5 and CYP2W1
Source: Vet Sci. 2026 May 14;13(5):476. doi: 10.3390/vetsci13050476 (PMC13211710; doi:10.3390/vetsci13050476)
Supplement: Supplementary file 1 [file vetsci-13-00476-s001.zip › supplementary tableS1.pdf]

Table S1. The ingredients of broiler basal diet.

Ingredients	Percentage
corn	57.12%
soymeal	27.34%
cottonseed meal	3%
oil	2.82%
DDGS	3%
corn gluten meal	2%
L-lysine	0.64%
DL-methionine	0.2%
calcium hydrogen phosphate	1.26%
stone powder	1.6%
salt	0.35%
choline chloride	0.2%
additive	0.47%

Notes: The additive includes Vitamin A 8000 IU, Vitamin D3 1000 IU, Vitamin E 20 mg, Vitamin K3 0.5 mg, Vitamin B1 2.0 mg, Vitamin B2 8 mg, Vitamin B6 3.5 mg, Vitamin B12 0.01 mg, Pantothenic acid 10 mg, niacin 35 mg, folacin 0.55 mg, biotin 0.18 mg, choline 1300 mg, Cu 8 mg, Fe 80 mg, Zn 80 mg, Mn 80 mg, I 0.7 mg, and Se 0.15 mg.
