# Supplementary material for: Ferulic Acid Alleviates the Hepatotoxicity of Aflatoxin B1 on Broilers by Conjugating and Down-Regulating Chicken CYP1A5 and CYP2W1
Source: Vet Sci. 2026 May 14;13(5):476. doi: 10.3390/vetsci13050476 (PMC13211710; doi:10.3390/vetsci13050476)
Supplement: Supplementary file 1 [file vetsci-13-00476-s001.zip › supplementary tableS2.pdf]

Table S2. The nutritional levels of broiler basal diet.

Nutritional levels	Content
metabolic energy	3000 kcal/kg
crude protein	21%
digestible lysine	1.28%
digestible methionine	0.61%
digestible methionine+cystine	0.92%
digestible threonine	0.84%
digestible tryptophan	0.23%
calcium	0.92%
available phosphorus	0.37%
