# Supplementary material for: Ferulic Acid Alleviates the Hepatotoxicity of Aflatoxin B1 on Broilers by Conjugating and Down-Regulating Chicken CYP1A5 and CYP2W1
Source: Vet Sci. 2026 May 14;13(5):476. doi: 10.3390/vetsci13050476 (PMC13211710; doi:10.3390/vetsci13050476)
Supplement: Supplementary file 1 [file vetsci-13-00476-s001.zip › supplementary tableS3.pdf]

Table S3. The raw data of broiler chickens' body weight gain in different groups.

	Groups					
	**C group**	**AFB1 group**	**L group**	**M group**	**H group**	**FA group**
	1.254	0.974	1.074	0.943	1.212	0.837
	1.414	1.009	1.008	1.067	1.208	1.355
	1.148	1.062	1.109	1.230	1.260	1.455
	1.308	0.986	1.007	1.221	1.550	1.538
	1.317	0.987	1.062	1.218	1.082	1.422
	1.454	0.863	0.999	1.110	1.384	1.250
**Body weight gain (g)**	1.128	0.691	0.974	1.075	1.142	1.331
	1.186	1.078	1.168	1.140	1.439	1.300
	1.368	0.883	0.863	1.179	1.122	1.472
	1.183	0.948	0.691	1.301	0.940	1.590
	1.183	_	1.152	1.145	1.015	1.623
	1.149	_	1.197	0.873	_	1.386
	1.431	_	1.118	_	_	1.528
	_	_	0.843	_	_	_
