# Supplementary material for: Ferulic Acid Alleviates the Hepatotoxicity of Aflatoxin B1 on Broilers by Conjugating and Down-Regulating Chicken CYP1A5 and CYP2W1
Source: Vet Sci. 2026 May 14;13(5):476. doi: 10.3390/vetsci13050476 (PMC13211710; doi:10.3390/vetsci13050476)
Supplement: Supplementary file 1 [file vetsci-13-00476-s001.zip › supplementary tableS4.pdf]

**Table S4.** The raw data of AFB1-DNA concentration in broiler livers and AFB1-ALB concentration in broiler serum.

| | Groups | | | |
|---|---|---|---|---|
| | **AFB1 group** | **L group** | **M group** | **H group** |
| **AFB1-DNA adduct concentration in liver (ng/mL)** | 3.680689007 | 3.174392212 | 2.864373943 | 2.379554779 |
| | 3.87741377 | 3.101303579 | 2.683269243 | 2.723396943 |
| | 3.436180857 | 2.636497966 | 2.415691868 | 2.361689302 |
| | 3.644721881 | 3.10697481 | 2.441760103 | 2.521088927 |
| | 3.621502145 | 3.011476141 | 2.597355568 | 2.36691625 |
| | 3.468505212 | 3.038212137 | 2.581682686 | 1.848851298 |
| **AFB1-ALB adduct concentration in serum (ng/mL)** | 0.250492055 | 0.681291926 | 0.139350047 | 0.045153782 |
| | 2.187733411 | 0.073342643 | 1.177364869 | 0.063875783 |
| | 0.353502843 | 0.139976288 | 0.20217994 | 0.070724381 |
| | 1.720842638 | 0.045038232 | 0.075053604 | 0.273030436 |
| | 0.108059788 | 0.332898769 | 0.08997057 | 0.073837602 |
| | 0.269123569 | 1.575757756 | 0.055586334 | 0.046199734 |
| | 0.127683158 | 0.91930285 | 0.479527215 | 0.049819216 |
| | 1.633256495 | 0.292758935 | 0.147478119 | 0.228159694 |
| | 0.097506244 | 0.037984721 | 0.092585524 | 0.037895127 |
| | 1.748730167 | 0.055940941 | 0.9167025 | 0.045009451 |
| | 2.331039955 | 0.143357613 | 0.243221881 | 0.040159025 |
| | 0.376409445 | 1.103593664 | 0.039334421 | 0.037374579 |
| | 1.087883819 | 1.225257807 | 0.038036005 | 0.066499192 |
| | 1.384642081 | 0.079290583 | 0.046624488 | 0.037098235 |
| | 1.333076618 | 0.628786093 | 0.095423594 | 0.039707727 |
| | 1.000665486 | 0.488971921 | 0.041306349 | 0.037614919 |
