# Supplementary material for: Ferulic Acid Alleviates the Hepatotoxicity of Aflatoxin B1 on Broilers by Conjugating and Down-Regulating Chicken CYP1A5 and CYP2W1
Source: Vet Sci. 2026 May 14;13(5):476. doi: 10.3390/vetsci13050476 (PMC13211710; doi:10.3390/vetsci13050476)
Supplement: Supplementary file 1 [file vetsci-13-00476-s001.zip › supplementary tableS5.pdf]

Table S5. The raw data of serological tests and liver biochemical tests.

	Groups					
	C group	AFB1 group	L group	M group	H group	FA group
AST level in serum (IU/L)	20.10113	57.74287	24.86333	14.72128	15.7467	14.88663
	14.14616	58.73176	23.09274	14.58764	14.1396	15.32379
	24.57069	43.0465	25.32569	14.78369	17.21437	14.51552
	21.58654	53.17371	20.14834	14.5295	14.23213	14.18122
	_	_	_	18.19206	14.94056	17.8129
ALT level in serum (IU/L)	12.16459	15.78295	13.27813	14.74765	13.91774	13.49302
	12.36421	17.53232	15.23658	14.9489	12.78521	11.17281
	11.82184	15.46448	15.08224	13.69238	14.62225	11.92877
	11.87281	16.25992	13.95879	13.53485	14.56932	13.22657
ALP level in serum (U/L)	89.58976	117.7349	109.0216	105.5737	103.0951	102.2562
	89.37049	115.5518	107.4963	105.1765	100.1462	90.24437
	92.50374	115.5264	112.2247	102.5263	91.95717	91.44555
	_	_	114.1695	106.4889	89.09085	_
γ-GT level in serum (U/L)	21.27999	37.12	24.32002	24.68001	24.07998	14.96001
	21.87999	36.27998	25.8	19.95999	20.64003	17.28002
	21.72001	39.83997	22.76	23.48	16.88	15.32
	17.67999	40	29.68	25.80002	20.51998	13.71997
TBA level in serum (μmol/L)	1.933533	10.97432	4.122785	3.997128	3.768878	2.261328
	2.08459	9.977337	6.103398	4.810913	1.548338	2.404834
	1.910875	9.439206	3.602719	2.945619	1.925981	3.157098
	2.197884	7.365963	4.609634	3.587611	1.938726	2.190762
	2.590954	_	_	2.327669	3.851963	3.469184
TG level in serum (mmol/L)	0.3252691	0.8236083	0.4062839	0.3706817	0.3359303	0.4254423
	0.4170169	1.052968	0.773798	0.4482931	0.2942286	0.2971605
	0.3417221	0.8539159	0.4065197	0.53633	0.3127626	0.343188
	0.4112251	0.8318913	0.4552434	0.4459763	0.3925268	0.2700609
GST activity in liver (U/gprot)	8.752635087	7.019707111	10.33804142	9.923304127	10.82210993	12.23543222
	6.637289018	7.155314754	10.45451471	11.07570087	10.71060008	10.15796077
	8.458131612	8.081493549	9.472223656	10.43488859	11.92249613	11.30384789
	7.944110736	7.848209251	9.351363678	10.0708	11.15173538	10.53476689
	9.678250196	4.298972214	11.95552382	10.12096982	12.36297217	11.05800194
	8.744850332	6.821928267	10.65451653	11.2081939	9.94049859	10.27954832
	9.131812032	6.870937524	_	11.04775204	_	11.83645556
	9.940174538	_	_	_	_	_
SOD activity in liver (U/gprot)	1054.212173	886.6916638	832.8086029	865.1620598	996.698349	1309.667312
	1089.585432	848.2436173	923.0488346	900.9939463	999.5331411	1346.684197
	1195.73529	865.6066009	860.4251554	831.3119956	1062.597235	1270.953282
	1199.229637	855.9811374	862.2671868	922.4659561	1019.609575	1062.889832
	1269.629198	784.6950669	832.7861542	985.0357736	_	1364.571788
MDA	3.469624868	6.596792019	3.341052889	3.095924728	2.803795719	3.320170081

**concentration in liver (μmol/gprot)**	3.642655831	5.948750656	3.46327939	3.223660329	2.090170907	2.988641367
	2.487367228	6.598223005	3.666854282	2.620836796	2.1856913	3.569911184
	3.159119018	6.372717217	2.888627459	3.405945447	3.987893767	2.393355593
	3.469624868	5.955181450	3.349794144	2.500364024	3.689672525	3.528772178
	3.642655831	5.591556178	3.412315399	3.785420446	4.092840289	2.930298145
	2.487367228	5.748995411	3.00127098	4.392191695	_	3.120170081
	_	_	3.867725781	3.529311514	_	_
**ROS concentration in liver (RFU)**	18638	39100	13377	7874	8106	8427
	16503	51360	13505	8060	8003	7461
	18159	49250	17620	7305	8081	8506
	19767	39032	10743	9027	8241	7265
	15238	40389	14523	7681	7331	7895
