# Supplementary material for: Ferulic Acid Alleviates the Hepatotoxicity of Aflatoxin B1 on Broilers by Conjugating and Down-Regulating Chicken CYP1A5 and CYP2W1
Source: Vet Sci. 2026 May 14;13(5):476. doi: 10.3390/vetsci13050476 (PMC13211710; doi:10.3390/vetsci13050476)
Supplement: Supplementary file 1 [file vetsci-13-00476-s001.zip › supplementary tableS6.pdf]

Table S6. The raw data of CYP450 proteins expression in endoplasmic reticulum.

	**Groups**					
	**C group**	**AFB1 group**	**L group**	**M group**	**H group**	**FA group**
**The grayscale ratio of CYP1A5/ β -actin**	0.96028	1.28025	1.56621	1.17701	1.16913	0.59391
	1.07123	1.4843	1.41586	0.78982	1.0406	0.38511
	0.86488	1.51961	1.08098	0.94154	0.73606	0.65091
**The grayscale ratio of CYP2A6/ β -actin**	0.79867	1.0604	0.89234	0.79234	0.28402	0.52253
	0.83665	1.64541	0.92543	0.42543	0.29074	0.40312
	0.93002	1.47681	0.91681	0.61681	0.36031	0.30635
**The grayscale ratio of CYP2W1/ β -actin**	0.85554	1.27211	0.61822	0.8022	0.68272	0.61923
	0.76452	1.10663	0.92448	0.65259	0.59878	0.67668
	1.1282	1.33106	0.72918	0.51552	0.53198	0.70968
**The grayscale ratio of CYP3A4/ β -actin**	0.77677	1.14398	0.97705	0.5515	0.48837	0.60296
	1.00498	1.34469	0.73551	0.53062	0.4326	0.36427
	0.87865	0.99986	1.00562	0.52983	0.69932	0.56176
