# Supplementary figures and images for: Ferulic Acid Alleviates the Hepatotoxicity of Aflatoxin B1 on Broilers by Conjugating and Down-Regulating Chicken CYP1A5 and CYP2W1
Source: Vet Sci. 2026 May 14;13(5):476. doi: 10.3390/vetsci13050476 (PMC13211710; doi:10.3390/vetsci13050476)

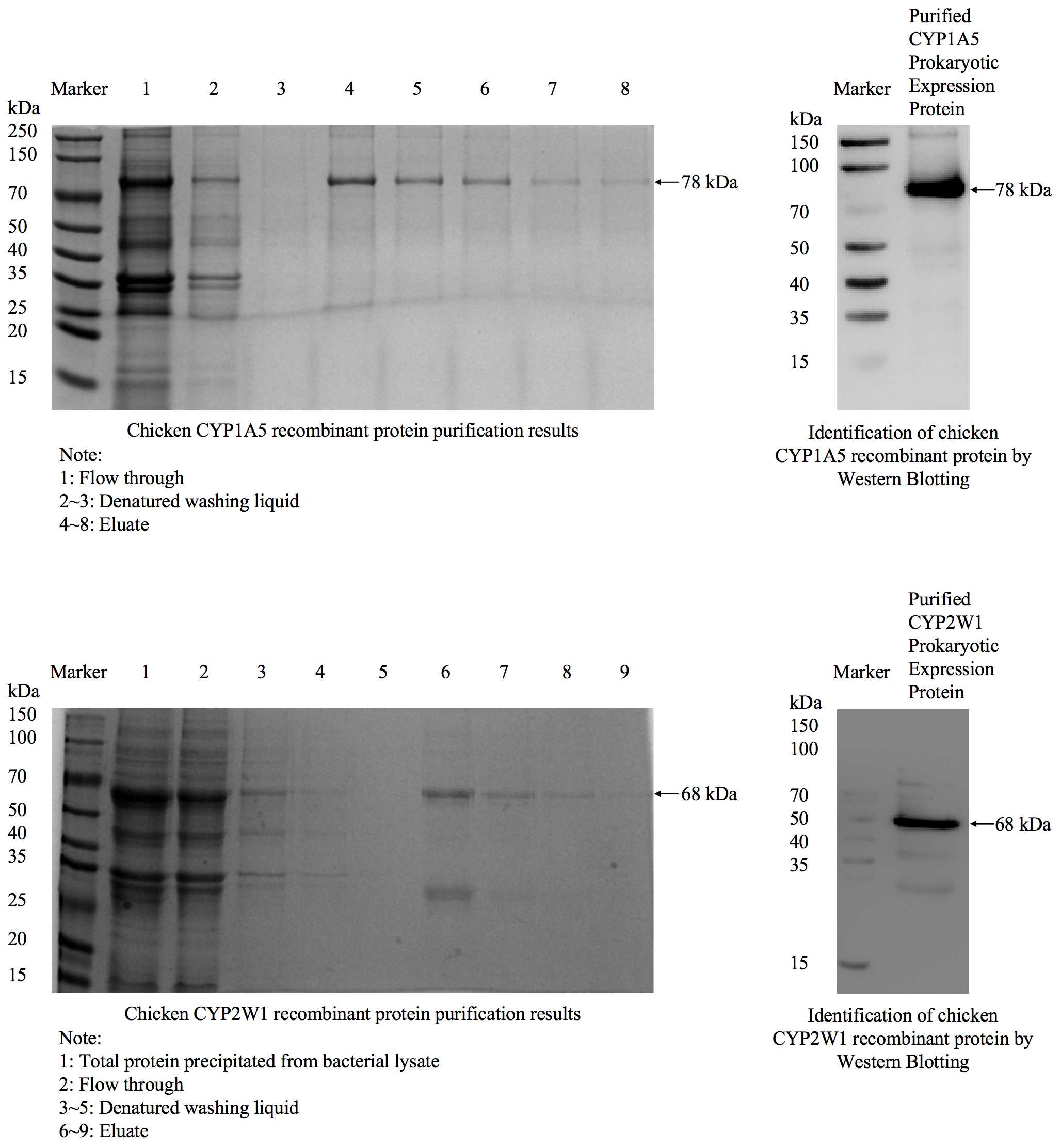

Supplement: Supplementary file 1 [file vetsci-13-00476-s001.zip › supplementary figureS1.jpg]

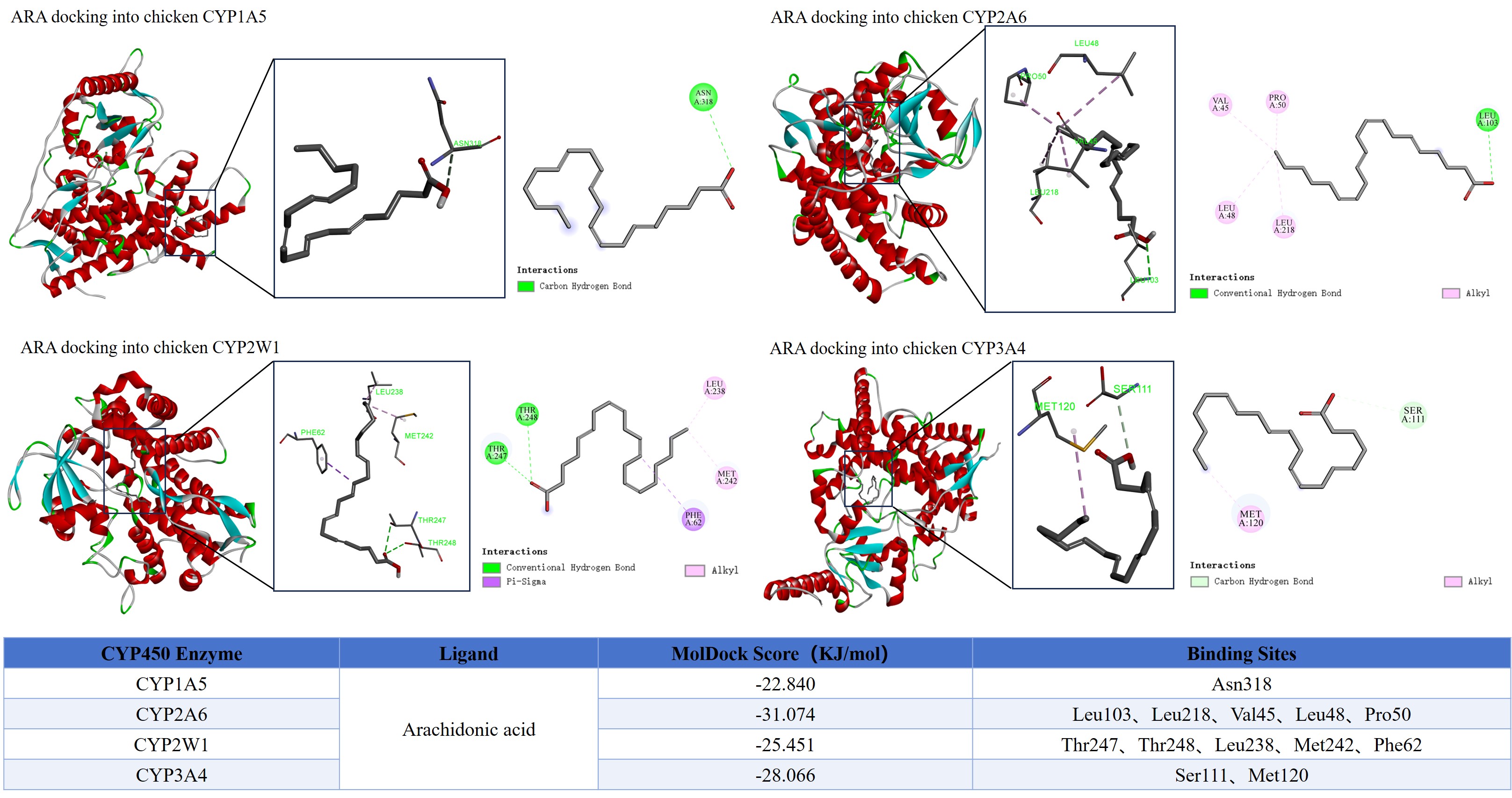

Supplement: Supplementary file 1 [file vetsci-13-00476-s001.zip › supplementary figureS2.jpg]

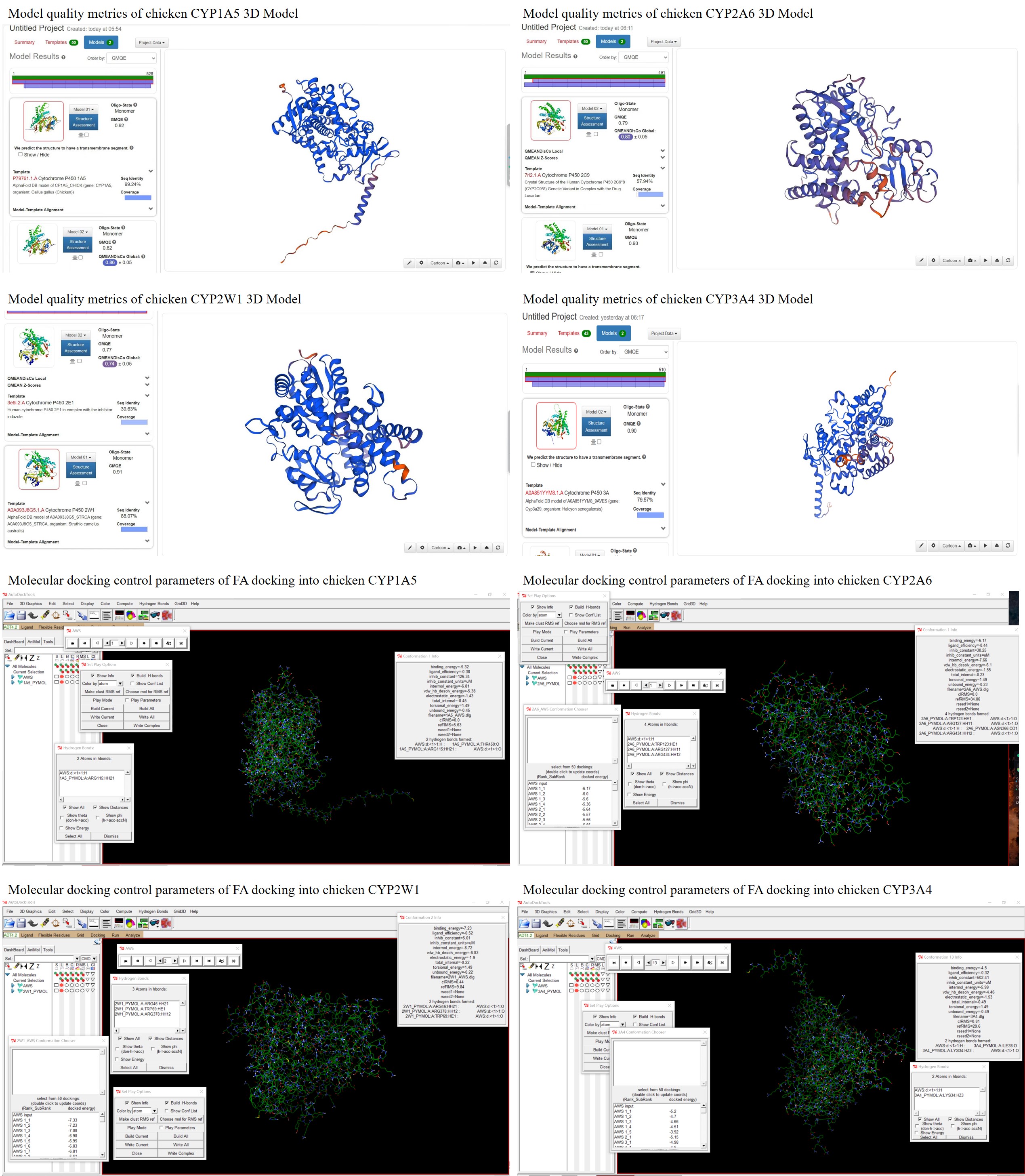

Supplement: Supplementary file 1 [file vetsci-13-00476-s001.zip › supplementary figureS3.jpg]
